# Legacy effects override soil properties for CO_2_ and N_2_O but not CH_4_ emissions following digestate application to soil

**DOI:** 10.1111/gcbb.12688

**Published:** 2020-04-27

**Authors:** Maria Chiara Rosace, Fabio Veronesi, Stephen Briggs, Laura M. Cardenas, Simon Jeffery

**Affiliations:** ^1^ Department of Crop and Environment Sciences Harper Adams University Newport UK; ^2^ Innovation for Agriculture Stoneleigh Park Warwickshire UK; ^3^ Sustainable Agriculture Sciences Department Rothamsted Research Devon UK

**Keywords:** carbon dioxide, digestate, legacy effects, methane, nitrous oxide, soil gas flux

## Abstract

The application of organic materials to soil can recycle nutrients and increase organic matter in agricultural lands. Digestate can be used as a nutrient source for crop production but it has also been shown to stimulate greenhouse gas (GHG) emissions from amended soils. While edaphic factors, such as soil texture and pH, have been shown to be strong determinants of soil GHG fluxes, the impact of the legacy of previous management practices is less well understood. Here we aim to investigate the impact of such legacy effects and to contrast them against soil properties to identify the key determinants of soil GHG fluxes following digestate application. Soil from an already established field experiment was used to set up a pot experiment, to evaluate N_2_O, CH_4_ and CO_2_ fluxes from cattle‐slurry‐digestate amended soils. The soil had been treated with farmyard manure, green manure or synthetic N‐fertilizer, 18 months before the pot experiment was set up. Following homogenization and a preincubation stage, digestate was added at a concentration of 250 kg total N/ha eq. Soil GHG fluxes were then sampled over a 64 day period. The digestate stimulated emissions of the three GHGs compared to controls. The legacy of previous soil management was found to be a key determinant of CO_2_ and N_2_O flux while edaphic variables did not have a significant effect across the range of variables included in this experiment. Conversely, edaphic variables, in particular texture, were the main determinant of CH_4_ flux from soil following digestate application. Results demonstrate that edaphic factors and current soil management regime alone are not effective predictors of soil GHG flux response following digestate application. Knowledge of the site management in terms of organic amendments is required to make robust predictions of the likely soil GHG flux response following digestate application to soil.

## Graphical abstract

A pot experiment was set up to evaluate N_2_O, CH_4_ and CO_2_ fluxes from cattle‐slurry‐digestate–amended soils. Digestate stimulated emissions of the greenhouse gases (GHGs) compared to controls. The legacy of previous soil management was a key determinant of CO_2_ and N_2_O while edaphic variables, in particular texture, were the main determinant of CH_4_ flux from soil following digestate application. Results demonstrate that edaphic factors and current soil management regime alone are not effective predictors of soil GHG flux response following digestate application and knowledge of the site management is required to predict soil GHG flux response following digestate application to soil.

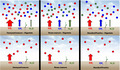

## 1 INTRODUCTION

Anaerobic digestion (AD) has potential benefits for the environment since it supports waste management, nutrient recycling and climate change mitigation though the substitution of fossil fuels (Møller, Boldrin, & Christensen, 2009; Möller & Stinner, 2009; Stinner, Möller, & Leithold, 2008). The main products of AD are biogas, which consists mainly of methane (CH_4_; Nkoa, 2014) and digestate, a high water content mixture composed of partially degraded organic matter (OM), microbial biomass and inorganic compounds (Appels et al., 2011).

Digestate application can reduce synthetic fertilizer use by recycling nutrients and improving the OM content in agricultural lands (Pezzolla et al., 2012). It has been shown to be an effective fertilizer for crop production (Alburquerque, Fuente, Campoy, et al., 2012; Alburquerque, Fuente, Ferrer‐Costa, et al., 2012; Barbosa, Nabel, & Jablonowski, 2014; Koszel & Lorencowicz, 2015; Möller & Müller, 2012) as it can increase the content of both macro and micronutrients in soil and plants (Chiew, Spångberg, Baky, Hansson, & Jönsson, 2015; Koszel & Lorencowicz, 2015). Nutrients in digestate, such as N, K and Ca are also provided in more plant available forms compared to the feedstock from which it was produced (Barbosa et al., 2014; Walsh, Jones, Williams, & Edwards‐Jones, 2012). It also provides labile OM that can function as a substrate for the soil biota (Alburquerque, Fuente, Campoy, et al., 2012; Möller & Müller, 2012). Digestate use in agriculture can therefore lead to a reduction in use of fertilizers, with associated reductions in the energy usage, and hence carbon footprint, required for fertilizer manufacture (Alburquerque, Fuente, Campoy, et al., 2012).

While digestate provides a valuable source of nutrients for crop production, its environmental impact in terms of interactions with soil GHG flux remain poorly understood (Verdi et al., 2019). Nitrous oxide (N_2_O) can be emitted from soils following digestate application, affecting air quality and contributing to climate change (Nkoa, 2014). However, N_2_O emissions are generally lower when digestate is applied to soils compared to the application of undigested feedstock (Möller & Müller, 2012). Carbon dioxide (CO_2_) and CH_4_ soil fluxes are also generally reduced when applying digestate compared to the undigested feedstock (Maucieri, Nicoletto, Caruso, Sambo, & Borin, 2017; Möller & Stinner, 2009). This is likely because most of the labile carbon is turned into biogas through the AD process, resulting in a digestate with less substrate present and so a lower potential for formation and emission of these gases (Clemens, Trimborn, Weiland, & Amon, 2006). Nevertheless, an increase in CO_2_ emissions after fertilization has been observed probably because of intensive decomposition of organic carbon after the application of easily degradable OM (Pezzolla et al., 2012) but emissions of GHG (such as CO_2_ and CH_4_) from fields fertilized with digestate on total emission from agriculture remain relatively low (Czubaszek & Wysocka‐Czubaszek, 2018).

Soil fluxes of CO_2_, CH_4_ and N_2_O all result from the actions of microorganism in the soil, as well as interactions with soil micro‐ and mesobiota in the case of CO_2_ in particular. These fluxes can be affected by soil properties such as bulk density, porosity and pore connectivity, water filled pore space (WFPS) and soil temperature (Bouwman, 1990; Nóbrega et al., 2015). N_2_O and CH_4_ emissions generally increase as WFPS increases such that soils contain more anaerobic than aerobic microsites (Bateman & Baggs, 2005; Smith et al., 2003). Some studies indicate that maximum microbial activity occurs within soil at around 60% WFPS (Fichtner, Goersmeyer, & Stefan, 2019; Linn & Doran, 1984). Furthermore, both N_2_O and CH_4_ emissions are generally higher in sandy soils compared with clay soils (Cai et al., 1999), likely due to the increased pore sizes of such soils facilitating gaseous diffusion meaning that such gases are emitted to the atmosphere before microbes have the chance to interact with them. However, other studies have reported higher emissions in clay soils, likely due to the generation of more anaerobic microsites in clay soil compared to sandy soils. Observed differences likely arise due to differences in management practices such as tillage leading to different soil structural impacts (Rochette, Angers, Chantigny, & Bertrand, 2008). While some agricultural management practices are well known to affect soil GHG flux, such as N fertilizer addition, the importance of legacy effects arising from management in previous growing seasons are less well understood. A previous study investigated the impact of amendments on soil nutrient turnover and GHG emissions, also comparing the application of the anaerobically digested materials together with the raw slurry (Johansen, Carter, Jensen, Hauggard‐Nielsen, & Ambus, 2013). They found that digestate application increased the soil concentration of ${\text{NO}}_{3}^{-}$ and assumed that respiratory denitrification was responsible for most of the N_2_O emitted, possibly due to anaerobic conditions. A further study by Abraha, Gelfand, Hamilton, Chen, and Robertson (2018) investigated the legacy effects of nitrogen addition on N_2_O flux from annual crop and perennial grasslands. Nevertheless, the role and interactions between soil properties and the legacy effects of previous soil amendment practices on soil GHG fluxes following digestate application to soil remains unknown. As such, this study aims to test the hypothesis that soil fluxes of individual greenhouse gases (GHGs; i.e. CO_2_, CH_4_ and N_2_O) will be impacted more by soil properties than by previous soil management.

## 2 MATERIAL AND METHODS

### 2.1 Field experimental set‐up

In April 2017, a field experiment was set up at Norbury Park, Staffordshire, United Kingdom (52°47′43.9″N, 2°17′24.0″W). It consisted of 18 plots (200 × 6 m) of three treatments, with six replicates, set up in a completely randomized design. Treatments were: (a) standard practice (hereafter SP), amended with 150 kg/ha of N‐fertilizer; (b) farm yard manure (hereafter FYM); (c) green manure (hereafter GM) which were drilled with a 50/50 mix of fodder radish (*Raphanus sativus*) and vetch (*Vicia* sp.) seeded at 29 kg seed/ha.

The FYM was collected from indoor beef cattle fed on grass, maize and barley. The feed was amended with Mix30—high energy liquid feed. FYM was applied to the treatment plots at a rate equivalent to 40 t FYM/ha eq.; the manure was stored for less than a week prior to application in the field with a muck spreader. It was then disked in to incorporate on the same day in March 2017. Total FYM nitrogen was 2.2%; mineral nitrogen was 0.6%.

All plots, except for the GM plots, were drilled with Spring wheat (var. Mulika) at a seed rate of 150 kg/ha in April 2017, at a drill depth of 3–4 cm.

In May 2017, FYM plots were fertilized with 125 kg/ha of Nitram (34.5% N) N‐fertilizer (i.e. to the same level of available N as the SP plots). GM plots did not receive any fertilizer. In October 2017, the wheat crop was harvested, and glyphosate (‘Round up Biactive GL. 360 g glyphosate’ 1.5 L/ha in 200 L water) was applied to all plots, including the GM plots, which were not harvested.

**FIGURE 1:**
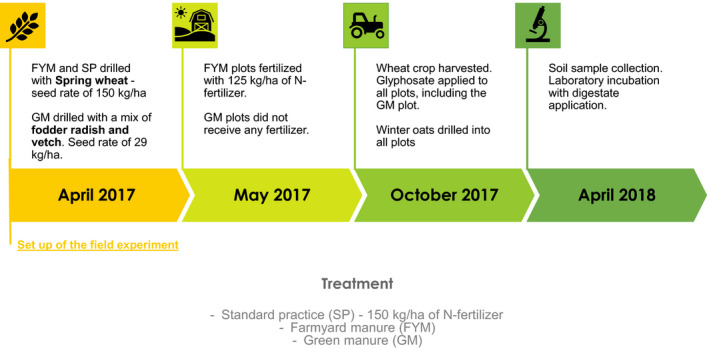
The timing of the application of the different treatments is reported, illustrating fertilizations, ploughings and cropping from April 2017 to April 2018

Subsequently, winter oats (var. Mascani) were drilled at 160 kg/ha eq., into all plots by direct drilling using a Weaving GD drill (Weaving Machinery) in October 2017 at a depth of 3–4 cm. This included direct drilling through the cover crop in the GM plots. Desiccated cover crops and straw residues were left on the plots as per the initial treatments. This whole process is summarized in Figure 1.

### 2.2 Laboratory incubation: Experimental design

In April 2018, soil samples were collected from each of the field experiment plots to a depth of approximately 10 cm using a spade. Soil texture fractions and types are reported in Table 1. Soil was sieved to pass 4 mm, and thoroughly mixed. Polypropylene pots (0.5 L) were packed with 300 g of soil from each plot to a bulk density of 1.3 g/cm^3^. Pots were watered to reach 60% WFPS, to replicate microbiallyactive but largely aerobic soils such as soils may be when digestate is applied; in fact the heavy trailer commonly used for digestate application necessitates soils having a high load bearing capacity, which soils with a much higher WFPS do not have. Pots were maintained gravimetrically at that moisture content through the timeframe of the experiment. Duplicate pots were made for each of the 18 field plots to total 36 pots. The pots were placed in a completely randomized design and preincubated in a climate‐controlled room at 20°C for 10 days to allow soil gas fluxes to equilibrate after the disturbance of soil sampling and packing into pots.

**TABLE 1:** Soil analysis showing three key soil characteristics at the end of the experiment (±*SE*)

Treatment replicates (from north to south)	Soil fractions			Soil texture^a^
	Sand (%)	Silt (%)	Clay (%)	
Green manure	68.2	16.3	15.5	Sandy loam
Farm yard manure	66.5	17.2	16.3	Sandy loam
Standard practice	65.7	18.7	15.6	Sandy loam
Standard practice	62.9	19.6	17.5	Sandy loam
Farm yard manure	61.4	21.3	17.3	Sandy loam
Green manure	63.3	18.9	17.8	Sandy loam
Green manure	58.4	21.8	19.8	Sandy clay loam
Standard practice	57.7	22.2	20.1	Sandy clay loam
Farm yard manure	59.3	21.5	19.2	Sandy clay loam
Green manure	60.0	21.9	18.1	Sandy clay loam
Standard practice	56.5	24.1	19.4	Sandy clay loam
Farm yard manure	61.2	20.4	18.4	Sandy clay loam
Green manure	59.8	21.6	18.6	Sandy clay loam
Farm yard manure	61.5	21.6	16.9	Sandy loam
Standard practice	61.4	21.0	17.6	Sandy loam
Farm yard manure	64.2	19.0	16.8	Sandy loam
Green manure	61.9	19.9	18.2	Sandy clay loam
Standard practice	66.9	17.1	16.0	Sandy loam

^a^ Texture calculated using LandIS (2019) based on fractions analysed in the lab.

A digestate produced by a mesophilic AD process (33 ± 7°C) of a cattle slurry (a mixture of milking cow slurry and young stock slurry, which was collected postseparation. No straw was included) at Harper Adams University, was applied to the surface of half of the duplicate pots at a rate equivalent to 250 kg/ha of total N. This is representative of the maximum permissible level of N applied as part of an organic amendment to soil annually (WRAP, 2012) within a Nitrate Vulnerable Zone within the United Kingdom. The other half of duplicate pots were kept as controls with no digestate applied but with water equivalent to the amount added with the digestate.

A subsample of the digestate was analysed for pH, total solids, volatile solids (VS), ash, VFA/TA ratio (an indicator for assessing fermentation processes; Table 2).

**TABLE 2:** Digestate properties and characteristics

Properties	Values
pH	8.18
TS (%)	3.71
VS (% of TS)	65.14
VFA (mg/L)	2,540.00
TA mg_eq_ CaCO_3_/L	13,952.95
VFA/TA ratio	0.18
Ammonium (g/L)	2.31
Ammonium (g/kg TS)	8.44

Abbreviations: TA, total alkalinity; TS, total solids; VFA, volatile fatty acids; VS, volatile solids.

### 2.3 Gas flux measurement

Soil GHG fluxes (CO_2_, CH_4_ and N_2_O) were sampled from the headspace of each of the 36 pots (six per treatment) on Day 0, 1, 2, 4, 8, 16, 32 and 64, 1 hr after closing the pot with a lid, using 30 ml gas syringe. The content of each syringe was passed through pre‐evacuated 20 ml vials, with the excess gas vented to ensure samples were stored at atmospheric pressure. A further six empty bottles were closed and sampled at equal times between the soil‐sample collections, in order to allow correction of the sampled gases for the background values of the gases present in the air inside the laboratory during the sampling time. Gas samples were transferred to 20 ml pre‐evacuated glass vials and transported to the laboratory. The concentration of GHGs was determined by gas chromatography (Perkin Elmer Clarus 500 gas chromatograph fitted with a Turbomatrix 110 automated headspace sampler, and an Electron Capture Detector [ECD] set at 300°C for N_2_O analysis). The Clarus system is fitted with two parallel Perkin Elmer Elite PLOT‐Q megabore capillary columns (oven temp 35°C), enabling the simultaneous analysis of methane, carbon dioxide and nitrous oxide from the air samples. Methane and carbon dioxide being detected using a Flame Ionization Detector (FID), fitted with a methanizer (350°C), and nitrous oxide via an ECD (300°C).

Daily fluxes measured in parts per million (ppm) were first converted to mg CO_2_‐C hr^−1^ m^−2^, mg CH_4_‐C hr^−1^ m^−2^ and mg N_2_O‐N hr^−1^ m^−2^, considering a temperature of 20°C (i.e. 293.15 K) and *R* (gas constant) of 0.0821 atm L^−1^ mol K^−1^ (R*T = 24.067615) as follows:

$$\text{mg}\;{\text{CO}}_{2}\text{-}C\;{\text{hr}}^{-1}m^{-2}=\left(\frac{\frac{\text{ppm}}{h}*\text{total}\;\text{volume}\;\text{head}\;\text{space}*\text{atomic}\;\text{weight}}{24.067615}\right):0.00658993\;m^{2}:1,000.$$

Values were finally converted to cumulative fluxes assuming linearity of flux rate between each measurement day.

### 2.4 Soil analysis

Soil electrical conductivity and pH were measured using 1:5 mix of soil/water with a pH meter (model 3510; Jenway) and conductivity meter (model 4510; Jenway). Moisture and OM contents of soil were measured by drying samples at 105°C to determine the moisture content, followed by loss on ignition at 550°C for 4 hr (Table 3).

**TABLE 3:** Soil analysis showing three key soil characteristics at the end of the experiment (±*SE*)

	pH	EC (µS/cm)	OM (%)
SP + digestate	6.64 ± 0.2	231.83 ± 9.46	3.55 ± 0.05
GM + digestate	7.40 ± 0.18	263.67 ± 15.37	3.66 ± 0.12
FYM + digestate	6.77 ± 0.12	275.50 ± 18.63	3.82 ± 0.1
SP control	6.68 ± 0.16	90.88 ± 8.26	3.42 ± 0.03
GM control	7.42 ± 0.16	142.95 ± 12.66	3.59 ± 0.09
FYM control	6.77 ± 0.12	131.48 ± 10.98	3.69 ± 0.11

Abbreviations: EC, electrical conductivity; FYM, farm yard manure; GM, green manure; OM, organic matter; SP, standard practice.

At the end of the experiment, soil texture was measured for each of the 36 samples by sieving and sedimentation (ADAS, 1986; Table 1).

### 2.5 Statistical analysis

Statistical analysis was undertaken by using R version 3.5.1 (R Core Team, 2018). Soil pH, electrical conductivity (EC) and OM data were first analysed using a Shapiro test and a Bartlett test. Subsequently, a two‐way ANOVA, with Tukey's HSD used post hoc, was used to assess differences between the treatments and digestate versus no digestate application.

A mixed effect model was used to compare soil texture, soil amendment legacy and digestate application, to assess which of these parameters was the most important predictor of GHG emissions.

Digestate versus no digestate application, soil amendment legacy and soil texture were included as fixed‐effects variables while the sampling date (Day) was included as a random effect. The model was fitted using REML (restricted maximum likelihood estimation), a modification of maximum likelihood estimation that is more precise for mixed‐effects modelling (Baayen, Davidson, & Bates, 2008). Three different data sets were built for the three different gases (CO_2_, CH_4_ and N_2_O) and the results were interpreted separately for each. The R package ‘emmeans’ (estimated marginal means) was used for post hoc comparisons following each mixed effect model to compute contrasts (Russell, Henrik, Jonathon, Paul, & Maxime, 2018) between each combination of the factors using pairwise contrast and Tukey's method.

## 3 RESULTS

### 3.1 Soil chemical characteristics: pH, EC and OM

Soil pH was not significantly affected by the digestate application and no differences were found when comparing digestate‐amended samples with those that did not receive digestate application (*p* = .89). GM samples had overall a higher soil pH compared with FYM (*p* < .001) and SP (*p* < .001). Digestate‐amended soil treatments had a significantly higher EC than those without the digestate applied (*p* < .001) and overall SP samples exhibited lower EC values compared to GM (*p* < .01) and FYM (*p* < .001). No change was observed in the content of OM between the control pots and the digestate‐amended pots (*p* = .12) but soil from the FYM‐treated field plots had significantly higher OM content compared to SP‐plots (*p* = .01).

### 3.2 CO_2_ emissions

The legacy effects of the previous field amendments can be observed in cumulative flux differences between the controls without digestate (*p* < .001). Between Day 1 and Day 64, average soil flux rate increased across the controls between 5.5 and 23.4 mg CO_2_‐C hr^−1^ m^−2^ in SP, 6.2 and 17.2 mg CO_2_‐C hr^−1^ m^−2^ in GM and 8.9 and 27.2 mg CO_2_‐C hr^−1^ m^−2^ in FYM.

Following amendment of the digestate, CO_2_ emissions were initially stimulated before reducing, approximately 10‐fold, over the 64 days of the experiment. By treatment, fluxes increased to 310.1 mg CO_2_‐C hr^−1^ m^−2^ initially before reducing to 32.8 mg CO_2_‐C hr^−1^ m^−2^ at Day 64 in SP + digestate, to 295.7 reducing to 25.2 mg CO_2_‐C hr^−1^ m^−2^ in GM + digestate, and to 336 reducing to 36.7 mg CO_2_‐C hr^−1^ m^−2^ in FYM + digestate. The application of digestate significantly increased CO_2_ emissions compared with control pots with no digestate applied (*p* < .001). No significant impact of soil texture on CO_2_ flux was observed within the constraints of this experiment (*p* = .21).

**FIGURE 2:**
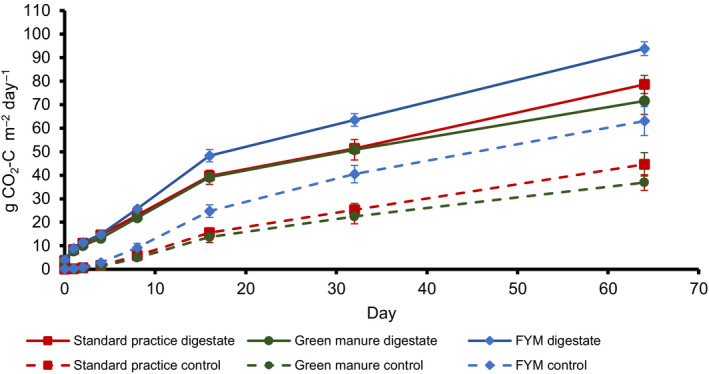
Cumulative CO_2_ emissions over the timeframe of the experiment showing both field treatments with digestate (solid markers) and the field treatments without digestate (i.e. controls; empty markers). Markers show means (*n* = 6). Bars show standard errors

Farm yard manure‐amended pots showed significantly higher CO_2_ emissions compared with GM treatment (*p* < .01) and SP treatment (*p* < .001). This was true for both digestate‐amended pots and for control pots. No significant differences were found between GM and SP treatments (*p* = .129) in either the control or digestate‐amended pots (Figure 2).

### 3.3 CH_4_ emissions

**FIGURE 3:**
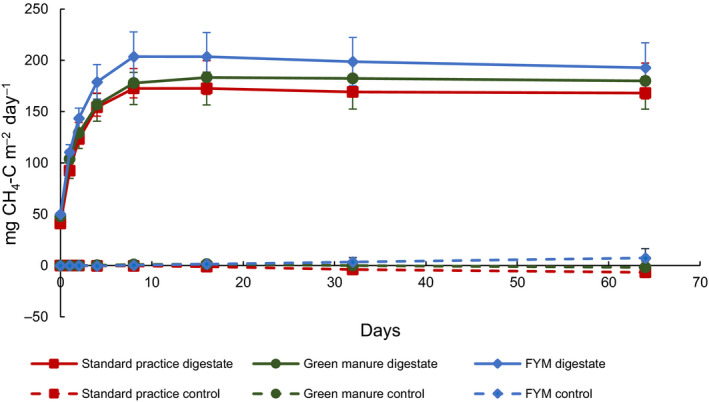
Cumulative CH_4_ emissions over the timeframe of the experiment showing both field treatments with digestate (solid markers) and the field treatments without digestate (i.e. controls; empty markers). Markers show means (*n* = 6). Bars show standard errors

Digestate application stimulates CH_4_ flux significantly (*p* < .001). Peak CH_4_ emissions were observed immediately after digestate application, which lasted for about a week before declining to no net emission thereafter. In digestate‐amended pots, CH_4_ emissions ranged from 3.4 mg CH_4_‐C hr^−1^ m^−2^ on Day 1 to 0 mg CH_4_‐C hr^−1^ m^−2^ by Day 8 in SP, from 4 mg CH_4_‐C hr^−1^ m^−2^ on Day 1 to 0 mg CH_4_‐C hr^−1^ m^−2^ by Day 16 in GM and from 4.2 mg CH_4_‐C hr^−1^ m^−2^ on Day 1 to 0 mg CH_4_‐C hr^−1^ m^−2^ by Day 8 in FYM. Methane fluxes from control pots with no digestate applied were not significantly different from zero (Figure 3). Emissions from FYM + digestate were significantly higher than SP + digestate (*p* = .03), but no significant differences were observed between GM + digestate and SP + digestate (*p* = .49). Soil texture significantly influenced CH_4_ emissions only when interacting with previous management practices (FYM) and digestate application (*p* < .001).

### 3.4 N_2_O emissions

**FIGURE 4:**
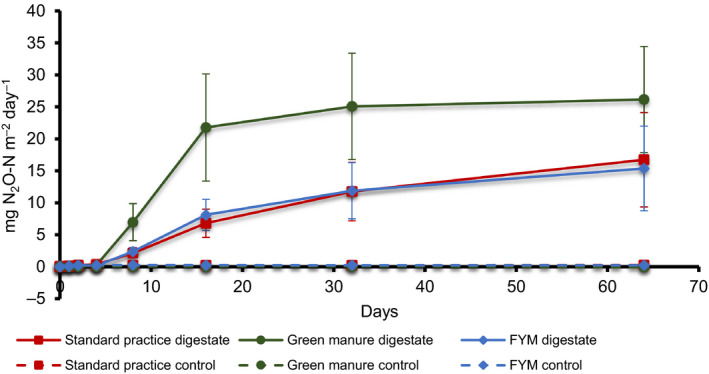
Cumulative N_2_O emissions over the timeframe of the experiment showing both field treatments with digestate (solid markers) and the field treatments without digestate (i.e. controls; empty markers). Markers show means (*n* = 6). Bars show standard errors

During the first 4 days of incubation, N_2_O emissions were not significantly different between any treatments (Figure 4) with values close to 0 net N_2_O emissions. A marginally significant increase was observed after Day 8, which was largest in the GM + digestate treatment (*p* = .045).

The GM + digestate treatment produced significantly higher cumulative N_2_O emissions (*p* < .001) compared with FYM + digestate and SP + digestate.

Soil texture did not significantly influence N_2_O emissions (*p* = .92) but a significant interaction between texture, digestate application and GM treatment was observed (*p* < .001). As for CH_4_ emissions, N_2_O flux was higher in digestate‐amended pots when the proportion of sand was lower (*p* = .06) but it was the legacy of previous amendment a year previously that was the strongest driver of increased N_2_O emissions following the addition of digestate (*p* < .001).

## 4 DISCUSSION

We hypothesized that soil GHGs fluxes following digestate application will be impacted more by soil properties than by previous soil management. This hypothesis should be accepted for CH_4_. However, the hypothesis should be rejected for CO_2_ or N_2_O. This means that, all else being equal, knowledge of the soil variables, such as those which may be present in soil maps or global soil databases, would be sufficient to allow effective prediction of the likely soil CH_4_ flux response to digestate application. However, it would not be sufficient to allow effective prediction of the likely soil CO_2_ and N_2_O flux response to digestate application. For these GHGs it would also be necessary to know the history of the previous season soil management at least, in terms of FYM application, or cropping versus cover cropping for GM, to more effectively predict the likely soil gas flux response to digestate application. This finding builds on work by Brenzinger, Drost, Korthals, and Bodelier (2018) who stated that ‘Future research should focus on the interrelation of plants, soil and microbes and their impact on the global warming potential (GWP) in relation to applied organic amendments’ to show that it is also necessary to know the history of management for at least the previous season to understand the GWP in relation to applied organic amendments.

**FIGURE 5:**
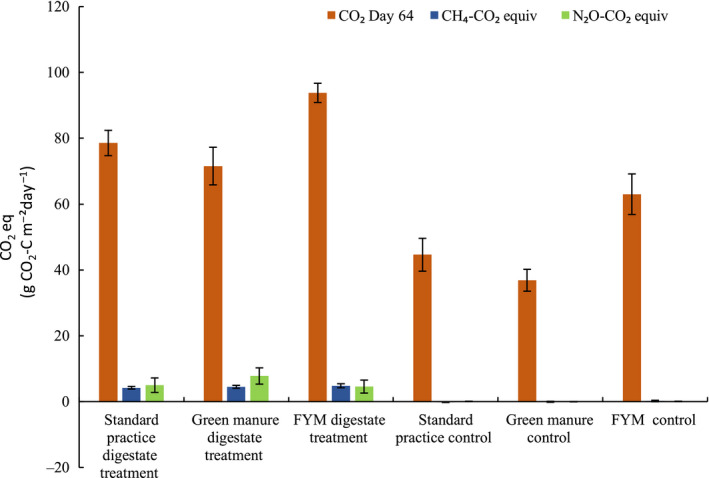
Cumulative emissions of the three gases (CO_2_, CH_4_ and N_2_O) as CO_2_‐equivalents at the end of the experiment (Day 64) on the basis of their global warming potential. Bars show standard errors. FYM, farm yard manure

The CO_2_‐equivalent emissions of the three gases in terms of their GWPs in a 100 year time horizon (Forster et al., 2007) are shown in Figure 5.

### 4.1 CO_2_ fluxes

CO_2_ soil fluxes occur in the absence of the amended digestate due to the ongoing decomposition of soil organic matter (SOM) in the pots (control samples, Figure 2).

Addition of the digestate stimulated CO_2_ fluxes from the FYM and GM soil beyond those stimulated by SP treatment. This indicates the presence of labile carbon in the digestate (Pezzolla et al., 2012) and suggests that the microbial communities in the FYM and GM treatments were primed (Zimmerman, Gao, & Ahn, 2011) to mineralize that labile carbon more readily than that in the SP treatment.

Apart from digestate application, two additional variables could have influenced the emission of CO_2_: physical soil properties (i.e. soil texture) or previous management practices (FYM, GM or SP). Soil texture at the Norbury Park sampling site changes between plots moving from north to south (Table 1), with sandy loam soils interspersed with sandy clay loam soil. Sand percentage range varied between 56% and 69% (Table 1) and was included as a fixed effect in the mixed model. While previous studies have reported soil texture to be a predictor of soil CO_2_ flux (e.g. Fiedler et al., 2017), no significant effect of soil texture was observed in this experiment (*p* = .21). Yang, Fan, and Jones (2018) reported significant impacts of texture on CO_2_ flux, even between very closely related soil taxa (i.e. loam, silty loam and silty clay loam). If greater variation in soil texture was present in the current study, it is possible that this may have influenced CO_2_ flux more significantly. This contrasts with CH_4_ and N_2_O, as discussed below, where soil texture did significantly affect both CH_4_ and N_2_O fluxes when interacting with previous management practices and digestate application.

Farm yard manure‐amended pots showed significantly higher CO_2_ emissions compared with GM treatment (*p* < .01) and SP treatment (*p* < .001). This was true for both digestate‐amended pots and for control pots. No significant differences were observed between GM and SP treatments (*p* = .129) in either the control or digestate‐amended pots.

It has been demonstrated that soil microbial diversity can be affected by fertilizer and organic amendment management practices (Lupwayi, Lafond, Ziadi, & Grant, 2012; Sradnick, Murugan, Oltmanns, Raupp, & Joergensen, 2013; Yu et al., 2015). FYM application may have increased the microbial biomass (Dhull, Goyal, Kapoor, & Mundra, 2004; Ghoshal & Singh, 1995) such that it was able to more quickly utilize new source of labile carbon (i.e. digestate) following application. Dhull et al. (2004) evaluated the short‐term effect of different doses of chemical fertilizers with and without organic amendment (i.e. FYM and GM). The study demonstrated that the application of organic amendments increased the microbial biomass C as well as soil respiration. There is evidence that fertilizer application and increasing inputs of crop residues increase SOM (Graham, Haynes, & Meyers, 2002) and microbial biomass carbon (Mahmood, Azam, Hussain, & Malik, 1997). Soil respiration activities display similar results as soil microbial biomass which is dependent on the organic C in the soil (Hofman, Dusek, Klanova, Bezchlebova, & Holoubek, 2004). Soil respiration can be used as a proxy for microbial activities in the soil. Ghoshal and Singh (1995) evaluated the changes in soil microbial biomass following applications of FYM in two annual cycles and demonstrated that the application of organic manure increased the microbial biomass.

Alternatively, a change in the microbial community structure may have been triggered by the application of the FYM such that the microbial community was more efficient at decomposing the applied digestate. Further studies and molecular analysis are necessary in order to investigate the importance of microbial abundance versus community structure to better understand the mechanism underlying this observed result. Nevertheless, the results show that the legacy of soil amendment 1 year before was a more important driver of differences than edaphic factors regarding CO_2_ emissions from soil following digestate application (Figure 2).

### 4.2 CH_4_ flux

Soil CH_4_ flux can be highly variable because methane microbial production and consumption can simultaneously occur in the soil within different microsites. CH_4_ is generally consumed in aerated soil, such as upland soils, when methanotrophic bacteria oxidize CH_4_ to CO_2_ while concurrently assimilating a large proportion of CH_4_‐C, often as much as half or more, into microbial biomass C (Mills et al., 2013). Conversely methanogenesis is an anaerobic process. CH_4_ usually produced in wetland areas, and areas subject to flooding as methanogenic activity is higher in soils with high water content (Le Hyaric et al., 2011).

Soil amendments can stimulate CH_4_ production. ChadwickPain and Brookman (2000) reported that over 90% of the total CH_4_ emissions occur during the first 24 hr following animal manures (i.e. pig slurry and dairy cow slurry) application. Here peak CH_4_ emissions were observed immediately after digestate application, which lasted for approximately 8 days before declining to no net emission thereafter (Figure 3). This result agrees with other studies that reported short‐term CH_4_ emissions after organic fertilizer application (e.g. Eickenscheidt, Freibauer, Heinichen, Augustin, & Drösler, 2014; Jones, Rees, Skiba, & Ball, 2005; Rees et al., 2004).

Also in agreement with previous studies (e.g. Smith et al., 2003), methane fluxes from control pots (i.e. field soil with no digestate) were not significantly different from zero.

Emissions from FYM + digestate were significantly higher than SP + digestate (*p* = .03), but no significant differences were observed between GM + digestate and SP + digestate (*p* = .49). This suggests that the GM treatment did not modify the soil environment such that it enriched or reduced the community of methanogenic microorganisms more than it did the community of methanotrophic microorganisms (Horz et al., 2002).

Higher CH_4_ fluxes were emitted by FYM + digestate with a lower percentage of sand. Methanogenesis is likely to occur in more poorly drained soil because of the higher presence of anaerobic microsites, which can then be exacerbated by digestate application that can further stimulate CH_4_ flux from those soils.

Applying digestate in wet or waterlogged soils can lead to higher GHG fluxes, including methane, due to the high soil moisture conditions that favour anaerobic microsites (Eickenscheidt et al., 2014). Reduced methane fluxes were observed when the sand percentage was higher, in agreement with previous studies on marshland soils (Le Mer & Roger, 2001) as well as in semi‐arid climate soils (Barton, Hoyle, Stefanova, & Murphy, 2016). This can be explained by soils with a lower proportion of sand (i.e. more clay) being less free draining, with smaller pores, and so have increased potential for more anaerobic microsites to be present where methanogenesis can occur.

### 4.3 N_2_O flux

N_2_O is mostly produced by microbial processes of nitrification and denitrification (Mojeremane, 2013; Wrage, Velthof, Beusichem, & Oenema, 2001) as well as other biochemical or chemical pathways (Nkoa, 2014) and processes as summarized by Butterbach‐Bahl, Baggs, Dannenmann, Kiese, and Zechmeister‐Boltenstern (2013). N_2_O emissions can be affected by soil pH and texture, moisture content, temperature and fertilizer application (Fiedler et al., 2017; Mojeremane, 2013; Stevens & Laughlin, 1998). Both nitrification and denitrification rates can increase in nitrogen fertilized systems because nitrogen provides a substrate suitable for N_2_O production. Similar to CH_4_, sandier soils generally show lower N_2_O emissions than clay soil due to the free draining nature of such soils meaning few anaerobic microsites are present (Skiba & Ball, 2006).

The low emissions observed initially were likely due to an initial phase of N immobilization induced by the OM application, followed by an N mineralization phase (Burger & Venterea, 2007; Morvan, Nicolardot, & Péan, 2006). It was expected that this phase would result in N_2_O emissions as the pots were maintained at 60% WFPS so the diffusion of O_2_ into the soil would be restricted. This would increase the proportion of anaerobic microsites compared to dry soil, thereby increasing N_2_O emissions compared to soil with lower WFPS (Bateman & Baggs, 2005). However, a 90% WFPS was not used for this experiment, as is sometimes used for experiments to stimulate N_2_O emissions (e.g. Sánchez‐García, Roig, Sánchez‐Monedero, & Cayuela, 2014), as we aimed to mimic aerobic soils that were suitable for trafficking, such as those where digestate would more usually be applied. It is unlikely that digestate would be applied to much wetter soils as they would not well support the heavy trailer commonly used for digestate application.

The GM field treatment was drilled with a mix of fodder radish and vetch. Vetch, as a legume, can fix N and so enrich the soil's N content (Campiglia, Mancinelli, Radicetti, & Caporali, 2010; Eichner, 1990; Ranells & Wagger, 1996). As such, the presence of this legume in the GM plots, which was not present in the other field treatments, may explain why N_2_O emissions from GM + digestate were significantly higher than FYM + digestate and SP + digestate. It is likely that the presence of the vetch interacted with the soil microbial community responsible for N cycling within the soil such that it was primed to cycle N more rapidly following the input of more N into the system (Lupwayi, Kennedy, & Chirwa, 2011; Sharma, Aneja, Mayer, Munch, & Schloter, 2005)—in this case the N that was present in the digestate. However, this hypothesis would require further research to test. That N_2_O flux was highest in GM + digestate which shows that edaphic factors alone are not sufficient descriptors of the likely GHG response of soil but the legacy of previous soil amendments also plays a role in determining GHG emission, particularly N_2_O and CO_2_ fluxes, following digestate application.

### 4.4 Other variables

Soil pH did not differ significantly between the soil amended with digestate and unamended soils. Long‐term studies on the impact of digestate to soil chemical properties are rather limited (Nabel, Schrey, Poorter, Koller, & Jablonowski, 2017). Due to the alkaline pH of the digestate itself (i.e. pH 8.18; Table 2) an increase in the soil pH could have been expected following application of digestate. However, digestate can also contain various acidic compounds (e.g. precipitation of carbonates as CaCO_3_; e.g. Makádi, Tomócsik, & Orosz, 2012; Möller & Müller, 2012), and soil itself can have a buffering effect for pH. These factors combined such that no significant effect of digestate on pH was observed within the constraints of this experiment. Regarding soil EC, digestate‐amended soil treatments had significantly higher values (*p* < .001), suggesting an increased concentration of ions in the soil from the digestate (Voelkner et al., 2015).

Overall, SP samples had lower EC values compared to GM (*p* < .01) and FYM (*p* < .001), which may be explained by the inputs of nutrients and salts contained in the manure and resulting from the decomposition of the GM (Lopes do Carmo, Botelho de Lima, & Silva, 2016).

Soil from the FYM‐treated field plots had significantly higher OM content compared to SP plots (*p* = .01). This agrees with other studies (e.g. Menšík, Hlisnikovský, & Kunzová, 2019) that have also shown that SOM content can increase following the application of manure and, overall, the use of organic amendments such as FYM or GM may improve the availability of nutrients and soil properties (Hargreaves & Warman, 2008). This change to the edaphic environment likely had consequences for the microbial communities present, changing their structure and thus impacting on soil functions and their interactions with the applied digestate.

Table 4 reports an overview of the variables investigated in this study that potentially impacted GHG fluxes.

**TABLE 4:** Results overview

GHG	Soil texture	Legacy effects	Digestate
CO_2_	—	***	***
CH_4_	*	—	***
N_2_O	*	**	***

Note

No effects detected —, low effects*, moderate effects**, strong effects***.

Abbreviation: GHG, greenhouse gas.

## 5 CONCLUSIONS

This study investigated the role of legacy effects of previous management practices on GHG emissions from soil following digestate application. Digestate significantly stimulated emissions of CO_2_, CH_4_ and N_2_O compared to controls with no digestate application. Higher CH_4_ fluxes were emitted by FYM‐amended soil with lower percentage of sand after digestate was applied. The legacy of previous amendments was found to not be a significant factor in determining CH_4_ flux following digestate application. However, the legacy of previous soil management was found to be the key determinant of CO_2_ and N_2_O fluxes. Edaphic variables did not have a significant effect on CO_2_ flux, but had for N_2_O fluxes.

For N_2_O flux, the legacy of previous soil management, particularly GM in this instance, interacted with edaphic factors to determine the soil N_2_O flux rate. The presence of vetch in the GM, together with digestate application and the lower sand percentage, stimulated N_2_O emissions after an initial phase of N immobilization. The critical period for N_2_O emissions was between 8 and 16 days after the digestate application. Ensuring digestate is applied at a time such that crops are actively growing following the N mineralization phase will maximize N uptake efficiency and minimize losses through N_2_O emissions and leaching.

This study demonstrates that edaphic factors and current soil management regime alone are not sufficient predictors of soil GHG flux response following digestate application. Therefore, it is essential to have knowledge of the amendment history of the site for at least the previous growing season in order to effectively predict the likely GHG emissions from soil following digestate application. Further studies and molecular analysis are necessary to investigate the microbial abundance and community structure and so better understand the mechanism underlying these observed results.

## CONFLICT OF INTERESTS

The authors declare that they have no known competing financial interests or personal relationships that could have appeared to influence the work reported in this paper.
